# Optimization of New Catalytic Topoisomerase II Inhibitors as an Anti-Cancer Therapy

**DOI:** 10.3390/cancers13153675

**Published:** 2021-07-22

**Authors:** Victor M. Matias-Barrios, Mariia Radaeva, Chia-Hao Ho, Joseph Lee, Hans Adomat, Nada Lallous, Artem Cherkasov, Xuesen Dong

**Affiliations:** The Vancouver Prostate Centre, Department of Urologic Sciences, University of British Columbia, 2660 Oak Street, Vancouver, BC V6H 3Z6, Canada; vmatiasbarrios@gmail.com (V.M.M.-B.); mradaeva@prostatecentre.com (M.R.); cho@prostatecentre.com (C.-H.H.); jlee@prostatecentre.com (J.L.); hadomat@prostatecentre.com (H.A.); nlallous@prostatecentre.com (N.L.); acherkasov@prostatecentre.com (A.C.)

**Keywords:** topoisomerase II, catalytic inhibitor, computer-aided drug design, cytotoxicity, DNA binding blocker

## Abstract

**Simple Summary:**

DNA topoisomerase II (TOP2) is a drug target for many types of cancers. However, clinically used TOP2 inhibitors not only kill cancer cells, but also damage normal cells, and can even give rise to other types of cancers. To discover new TOP2 inhibitors to more effectively treat cancer patients, we have applied computer-aided drug design technology to develop several TOP2 inhibitors that can strongly inhibit cancer cell growth but exert low side effects. Results of one exemplary compound are presented in this study. It shows several promising drug-like properties that can be potentially developed into anticancer drugs.

**Abstract:**

Clinically used topoisomerase II (TOP2) inhibitors are poison inhibitors that induce DNA damage to cause cancer cell death. However, they can also destroy benign cells and thereby show serious side effects, including cardiotoxicity and drug-induced secondary malignancy. New TOP2 inhibitors with a different mechanism of action (MOA), such as catalytic TOP2 inhibitors, are needed to more effectively control tumor growth. We have applied computer-aided drug design to develop a new group of small molecule inhibitors that are derivatives of our previously identified lead compound T60. Particularly, the compound T638 has shown improved solubility and microsomal stability. It is a catalytic TOP2 inhibitor that potently suppresses TOP2 activity. T638 has a novel MOA by which it binds TOP2 proteins and blocks TOP2–DNA interaction. T638 strongly inhibits cancer cell growth, but exhibits limited genotoxicity to cells. These results indicate that T638 is a promising drug candidate that warrants further development into clinically used anticancer drugs.

## 1. Introduction

Genomic DNA in a double-helical structure has to be precisely controlled in a topological homeostasis state when confronted by mechanical stresses arising from gene transcription, DNA replication, and chromosome segregation [1]. By transiently breaking the double-strand DNA and resealing it, DNA topoisomerase II (TOP2) enzymes provide a constant solution to prevent excessive DNA twists created by DNA manipulating protein factors, such as transcriptional factors and DNA polymerases [1]. TOP2 overexpression is associated with high proliferation indexes and poor prognosis in multiple types of tumors [2,3,4,5]. This creates an opportunity for anticancer therapy, since cancer cells are more sensitive to TOP2 inhibition than benign cells [1]. Human cells express two TOP2 genes, TOP2A and TOP2B [6,7]. TOP2A is deemed as an essential gene for cancer cell division, since its expression is specific for the S and M phases of cell cycling, and is required for DNA replication and disentangling interlinked chromosomes for mitosis [8,9]. In contrast, TOP2B is dispensable for cell viability [10,11], and its expression is independent of the cell cycle [6,7]. However, oncogenic transcriptional factors (e.g., androgen receptor (AR) [12,13,14]) require TOP2B to be recruited to their targeted promoters to initiate transcription to drive proliferative transcriptomes. Blocking both TOP2A and TOP2B could, therefore, serve as a double-bolt lock to effectively inhibit tumor growth and progression. 

TOP2 inhibitors can be classified into two types: TOP2 poisons and catalytic inhibitors [15,16]. Poisons (e.g., etoposide) stabilize the covalent TOP2–DNA cleavage complex, resulting in double-strand DNA breaks that can cause cell death [16,17]. However, this mechanism of action (MOA) is often associated with several serious side effects, such as cardiotoxicity and drug-induced secondary malignancy [18,19]. Patients treated with etoposide can develop acute myeloid leukemia (t-AML) [18] that had been demonstrated as attributing to TOP2B-mediated DNA breaks that disrupt mixed-lineage leukemia (MLL) and MLL-associated genes in human bone marrow cells [18]. Since TOP2B is expressed and remains active during the G1 phase of the cell cycle, even in nonproliferating benign cells, TOP2 poisons cannot differentiate cancer cells from benign cells when introducing genotoxic effects. 

TOP2 catalytic inhibitors, in contrast, do not induce the covalent TOP2–DNA cleavage complex, and thereby cause reduced DNA damage, but can inhibit TOP2 enzymatic activity through several different MOAs, such as preventing TOP2 proteins from interacting with DNA [20], blocking ATP from binding to TOP2 proteins [21], or stabilizing the noncovalent TOP2–DNA complex [22]. These catalytic inhibitors present low genotoxicity but remain effective in inhibiting cell division. These results provide a rationale that discovery and development of catalytic TOP2 inhibitors may be more selective to suppress cancer cells but result in no or low genotoxicity to non-dividing benign cells. Catalytic inhibitors are, therefore, sought after as prospective anti-tumor drugs to be applied alone or in combination with other clinically applied chemotherapies [23].

Our previous studies have applied computer-aided drug design (CADD) methodology to identify a druggable site at the TOP2–DNA interaction interface and developed a series of small molecule inhibitors to disrupt TOP2 from interacting with DNA [24]. The lead compound, T60 (PubChem CID:36589274), has shown potent inhibitory effects on both TOP2A and TOP2B enzymatic activities. It inhibits cancer cell proliferation, but has limited genotoxicity. Importantly, T60 has presented dual inhibitory effects to not only AR activity, but also AR-positive PCa cell growth, further underscoring that it is a promising drug candidate that warrants further drug development. However, it has poor solubility, hindering its effective delivery to tumor cells in both in vitro and in vivo studies. To improve the pharmacokinetic properties of T60 and further enhance its efficacy to inhibit TOP2 proteins, we have implemented a structure–activity relationship (SAR)-guided strategy to further optimize the scaffold of T60. Herein, we report a new series of T60 derivatives, among which, T638 has shown improved solubility and further enhanced metabolic stability. It has preserved effectiveness to block cancer cell proliferation. Because of its low cytotoxicity to cells, T638 can be administrated in much higher doses to achieve maximal tumor suppression.

## 2. Materials and Methods

### 2.1. In Silico Experiments

To generate new derivatives, the combinatorial builder package from Molecular Operating Software (MOE) was used [25]. To ensure that any modification of T60 will not change its affinity to TOP2 proteins, we have applied molecular docking using the GlideSP software [26] with default settings. The protein preparation before docking was performed in the same manner as our previous study [24]. The ADMET filtering was also conducted with ADMET prediction tool from SimulationPlus Inc (Lancaster, PA, USA) with the same parameters as we outlined [24]. To analyze the solubility of these T60 derivatives, we have averaged the outputs of three machine learning models—ESOL [27], Ali [28], and Silicos-IT [29] available from the SwissADME web platform [29]. We then calculated the free energy perturbation using FEP+ software from Schrödinger [30], since it had been demonstrated to be able to accurately and reliably predict ligand binding affinities [31,32]. To validate the choices by these software, we have run FEP+ on a test set of four compounds with known inhibitory activities (T606, T633, T634, and T627) [24]. Predicted delta G values correlated with the activity measured in decatenation and relaxation assays, proving the utility of the FEP+ calculations. These calculations were performed with all default settings.

### 2.2. Compounds

T60 derivatives were custom synthesized by Life Chemicals Ltd., with purity >90%. Upon receiving the compounds, the molecular masses were validated by LC/MS/MS.

### 2.3. Solubility 

Stock solutions of the tested compounds at 50 mM in dimethyl sulfoxide (DMSO) were diluted 50-fold in methanol (MeOH), Roswell Park Memorial Institute-1640 (RPMI1640), plus 5% charcoal-stripped serum (CSS) from Sigma-Aldrich (media) and phosphate-buffered saline (PBS), and vortexed at room temperature for 1 h at 800 rpm. The resulting solutions were centrifuged at 20,000× *g* for 5 min, and the saturated supernatants were diluted 40-fold in acetonitrile. The resultant solutions were subjected to LC/MS/MS to determine the solubility.

### 2.4. Microsomal Stability Assays

The in vitro microsomal stability assay was carried out using the mouse liver microsomal system (Xenotech, M1000), which was enriched with nicotinamide adenine dinucleotide phosphate and NADPH (BD Biosciences, San Jose, USA). Compounds at 1 uM concentration were incubated with a reaction mixture containing liver microsomal proteins (0.15 mg/mL) in 100 mM potassium phosphate buffer (pH7.4). The reaction was initiated by adding NADPH, followed by incubation at 37 °C. At various time points, one volume of reaction aliquot was taken and quenched with three volumes of acetonitrile containing 0.05% formic acid. The samples were then vortexed and centrifuged at 16,000 rpm for 10 min at 4 °C. The supernatants were collected and analyzed by LC/MS/MS to determine the remaining percentage and the half-life (t1/2) by integrating compound peaks and fitting the resulting AUC values and time points to a one-phase decay equation. Imipramine was used as a positive control with t1/2 of 24 min to confirm the activity of the microsomes.

### 2.5. Microscale Thermophoresis (MST) Assays

TOP2A(431-1193) protein purification has been reported by us [24]. Briefly, human TOP2A(431-1193) cDNA containing the DNA binding domain was cloned into the pET28a expression vector with an in-frame N-terminal 6xHis tag. It was transformed into BL21 (DE3) *E. coli* cells and induced by 0.5 mM IPTG for 16 h at 16 °C. Cells were lysed by sonication and protein purification was by chromatography with the Ni-NTA beads (Invitrogen), followed by a size exclusion chromatography (Superdex 200). MST assay was performed to determine the binding affinity of compounds to the purified TOP2A(431-1193). TOP2A(431-1193) protein samples were labeled with the red fluorescent dye NT647 using the Monolith NT Protein labeling kit RED-NHS amine-reactive (NanoTemper Technologies, München, Germany). A serial dilution of the inhibitors was prepared with 100% DMSO and subsequently mixed with the labeled fluorescent protein in the assay buffer (20 mM Tris pH 8.0, 100 mM NaCl, 0.1% NP40, 0.2 mM TCEP, and 0.1 mM PMSF) at the final concentrations of 0.015–500 uM. The final concentration of the protein was 5 nM. The labeled protein and small molecules mix were allowed to incubate in the dark at room temperature for 5 min before filling the capillaries. MST assays were performed with 5–20% LED/excitation power and medium MST power using premium capillaries for Monolith NT.115. The data for the estimation of Kd were analyzed using MO Affinity Analysis software from Nanotemper.

### 2.6. Fluorescence Polarization (FP) Assays

Fluorescent polarization assay was performed in Corning^®^ Low Volume 384-well Black Flat Bottom microplate. Briefly, the double-strand DNA oligo, 5ZRF (AGC CGA GCT GCA GCT CGG CT), labeled by Fluorescein amidite (FAM), was purchased from IDT (Coralville, IA, USA). Human TOP2(431-1193) protein at a concentration of 500 nM was incubated with 2 nM of 5ZRF oligo in a 20 µL FP reaction buffer containing 20 mM HEPES-NaOH, pH 7.4, 2 mM DTT, 3 mM MgCl2, 0.05% NP-40, and protease inhibitors. Triplicate reactions were transferred onto the microplate and incubated at room temperature for 20 min. Fluorescence polarization (FP) measurements were taken by Tecan Infinite F500 with the excitation wavelength of 485 nm and emission wavelength of 535 nm.

### 2.7. Other Routine Techniques

There are several routine biochemistry and molecular techniques, including K-DNA decatenation assays, DNA relaxation assays, ethidium bromide displacement assays, electrophoresis mobility shift assay (EMSA), DNA cleavage assays, cell proliferation, and cytotoxicity assays; immunoblotting assays had been reported previously by our lab [24]. Technical details of these assays are reported in the Supplementary Materials and Methods section attached to this manuscript. 

### 2.8. Statistics

We used the GraphPad Prism 5.01 software (GraphPad Software, San Diego, CA, USA) to perform statistical analysis. When comparing the differences between two groups, a Student *t*-test was used. When comparing differences among multiple groups, one-way ANOVA, followed a *t*-test, was applied. The levels of significance were set at *p* < 0.05 as *, *p* < 0.01 as **, and *p* < 0.001 as ***.

## 3. Results

### 3.1. Modification of the Scaffold of T60 Using CADD

**Figure 1:**
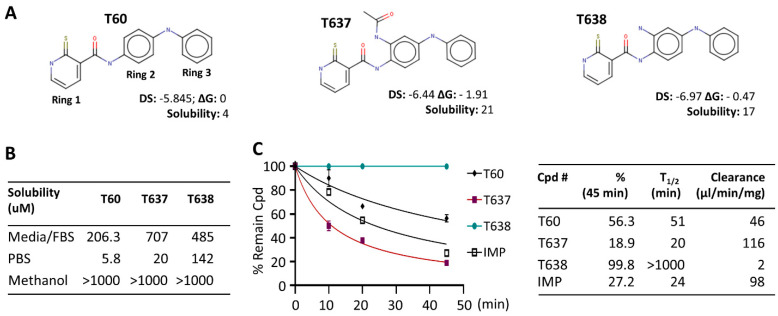
T60 derivatives have improved solubility and metabolic stability. (**A**) The structures of T60, T637, and T638 were designed by CADD. DS, docking score (the lower the better); ΔG, the free energy of binding estimated by FEP+ relative to T60; solubility, an average of three model predictions measured in μM/l; (**B**) The solubility of T60, T637, and T638 in cell culture media with 10% FBS, PBS, and methanol was determined by LC/MS/MS; (**C**) T60, T637, T638 microsomal stability was determined by incubating 1 uM of the compounds with 0.15 mg/mL mouse liver microsomes for 0, 10, 15, 20, and 45 min, with imipramine (IMP) as a positive control. The concentrations of each compound were measured by LC/MS/MS to calculate the clearance rates over the time course.

Our lead compound T60 from the previous study suffered from low solubility, making it difficult to detect maximum tumor suppression effects in in vitro and in vivo experiments [24]. Since solubility largely determines the compound concentrations exposed to target proteins in the tested systems, its improvement would significantly enhance the potency of tested compounds [33]. Therefore, we aimed to optimize the scaffold of T60 to achieve higher solubility while preserving its affinity to TOP2, using an SAR-guided strategy. We have introduced new functional groups only to ring 2 of T60 (Figure 1A), since our previous study has demonstrated that ring 2 tolerates modifications [24]. Furthermore, compounds T630, T633, and T634 with methyl or hydroxyl groups on ring 2 exhibited increased activities compared to T60 [24]. Importantly, this observation agreed with the presence of an adjacent cavity in the binding pocket, which could accommodate various functional groups on ring 2 and may, consequently, enhance ligand affinity [24]. A total of 1900 T60 derivatives with various functional groups on ring 2 were virtually designed using the combinatorial builder from MOE. We then performed molecular docking in the proposed binding pocket of T60 in TOP2A (PDB: 4FM9) to select compounds with docking scores higher than T60. Potentially toxic compounds were discarded with an ADMET predictor tool. These filtering procedures yielded 25 compounds. To estimate the solubility of the designed compounds, we employed machine learning models and found only 11 compounds were expected to have a higher solubility than T60. Furthermore, we have performed free energy perturbation (FEP) analyses to more precisely estimate the affinity of each T60 derivative to TOP2 protein. Compared to molecular docking, the FEP analysis provides a more accurate and reliable ranking of compound affinity to their target. However, it is more computationally intensive which is why we performed this analysis as the final filtering. To this end, three compounds showed lower docking scores, optimized ADMET index, improved solubility, and lower ligand-binding energy (FEP) in comparison to T60. We obtained two compounds, T637 and T638, that can be successfully synthesized by Life Chemical Inc. to allow us to perform biological validation.

### 3.2. T638 Has Improved Solubility and Microsomal Stability in Comparison to T60

Both T637 and T638 have improved solubility in aqueous solution and culture media compared to T60 (Figure 1). T60 has only 5.8 uM solubility in PBS buffer and ~200 uM in cell culture media containing 5% serum. When cells are growing in the culture media and treated with T60 at concentrations ≥50 uM for two days, T60 will form crystals, likely due to the altered pH values by the cells that induce T60 precipitation. However, these observations did not occur to T637 and T638. Their solubility is 20 uM and 142 uM in PBS and is 707 uM and 485 uM in the culture media, respectively. There was no crystal formation in cell cultures up to 100 uM. These improvements in solubility allow higher doses of compounds to be used to treat cancer cells to reach maximum tumor suppression. However, T637 has reduced microsomal stability, with a clearance rate of 116 ul/min/mg, compared to T60 at 46 ul/min/mg. In contrast, T638 showed exceptional microsomal stability, with a clearance rate of 2 ul/min/mg. In our assays, we have used imipramine (IMP), a clinically used oral drug for anti-depression, as a control. These results together indicated that T638 has improved solubility and metabolism stability in comparison to T60, thus it became a promising lead compound to validate its biological efficacy in suppressing TOP2 activity.

### 3.3. T638 Is an Inhibitor to Both TOP2A and TOP2B

**Figure 2:**
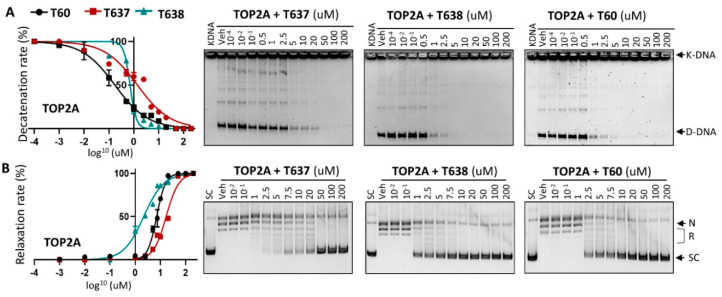
T637 and T638 are TOP2A inhibitors. (**A**) K-DNA decatenation assays and (**B**) supercoiled plasmid DNA relaxation assays are used to measure TOP2A enzyme activity in the presence of 0.01–200 uM T637 or T638. Three biological repeats were performed with each concentration of the compounds, and the densitometry of the D-DNA bands from (**A**) and the SC bands from (**B**) was used to establish an inhibition curve for IC_50_ calculation.

To ensure that improved solubility and metabolic stability of T638 did not affect its efficacy to inhibit TOP2 enzymatic activities, we have applied in vitro K-DNA decatenation and supercoiled plasmid DNA relaxation assays as we described [24]. We found that T638 inhibits TOP2A activity in a dose-dependent manner, with an IC_50_ at ~0.7 uM, similar to T60 at ~0.3 uM (Figure 2), but much lower than T637, with an IC_50_ at ~1.4 uM. However, T638 showed much stronger inhibition in the relaxation assays, with an IC_50_ at 2.1 uM in comparison with T60 and T637 at 4.7 uM and 16.8 uM, respectively.

**Figure 3:**
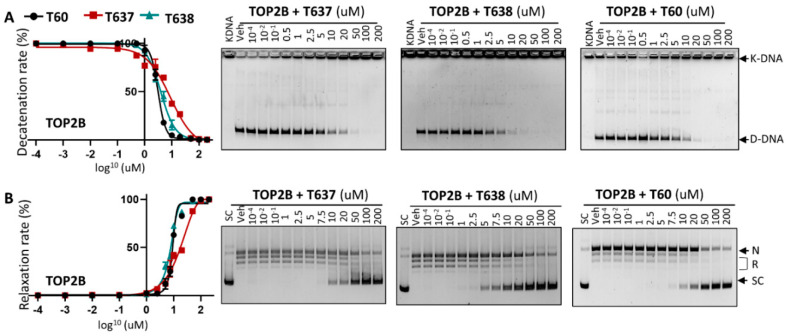
T637 and T638 are TOP2B inhibitors. (**A**) K-DNA decatenation assays and (**B**) supercoiled plasmid DNA relaxation assays are used to measure TOP2B activity in the presence of 0.01–200 uM T637 or T638. Three biological repeats were performed with each concentration of the compounds, and the densitometry of the D-DNA bands from (**A**) and the SC bands from (**B**) was used to establish an inhibition curve for IC_50_ calculation.

Since the docking pocket of T60 and its derivatives is identical between TOP2A and TOP2B, except for one amino acid (L722/F738, TOP2A/TOP2B) and this amino acid does not participate in T60 binding to TOP2, we next tested whether T638 has similar inhibitory effects to TOP2B. T638 inhibits TOP2B, with an IC50 of ~3.8 uM, similar to T60 at ~3.1 uM, but much stronger than T637, which has an IC50 of ~8.9 uM in K-DNA decatenation assays (Figure 3). In DNA relaxation assays, T638 inhibits TOP2B, with an IC50 of 7.5 uM, similar to T60 at about 8.9 uM, and much stronger than T637 with an IC50 at about 18.6 uM. These results confirmed that improved solubility and microsomal stability of T638 do not compromise its inhibitory effects to TOP2, and even show some improved TOP2 inhibition in the DNA relaxation assays.

### 3.4. T638 Is Not a DNA Intercalator but a TOP2 Binder

**Figure 4:**
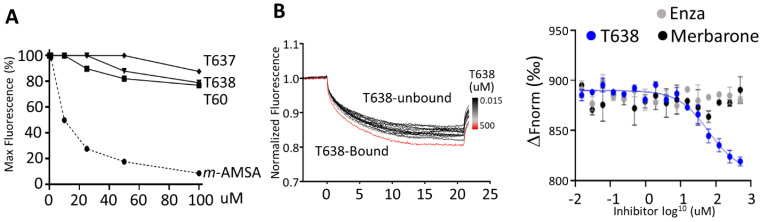
T638 does not intercalate with DNA but binds to TOP2 protein. (**A**) Ethidium bromide displacement assays were performed by mixing 100 µg/mL salmon sperm DNA with −/+2 µg/mL ethidium bromide and the indicated compounds. Reactions were subjected to fluorescence emission of 590 nm and excitation of 535 nm. Obtained signals were detected by using the Multimode Microplate Reader (Infinite F500). (**B**) MST assay was performed by incubating 5 nM of the purified human TOP2A(431-1193) labeled with the red fluorescent dye NT647 in the presence of vehicle or 0.015–500 µM of T638. Reactions were subjected to 5–20% LED/excitation power and medium MST power using premium capillaries. Obtained signals were detected by using the Monolith NT.115 system. The change in thermophoresis is expressed as the change in the normalized fluorescence (Δ*F*_norm_). MST assays were repeated with two control compounds, enzalutamide (Enza) and merbarone.

Since many TOP2 inhibitors, including those used in clinics (e.g., mitoxantrone and doxorubicin), are DNA intercalators that indirectly inhibit TOP2 activity, they will result in uncontrollable genotoxic and cytotoxic effects to not only cancer cells, but also benign cells. To exclude the possibility that T638 intercalates with DNA and, thereby, inhibits TOP2 activities, we performed ethidium bromide displacement assays (Figure 4A). The fluorescence induced by the ethidium bromide/DNA complex was reduced by increasing doses of the synthetic aminoacridine derivative amsacrine (m-AMSA) [34], a known DNA intercalator, but not T60, T637, or T638. These results showed that T60 does not intercalate with DNA, and suggested its inhibitory effects to TOP2 proteins are through direct interaction with TOP2 proteins.

To confirm that T638 binds TOP2 protein, we have applied MST assays (Figure 4B). The principle of this technique is that the directed movement of molecules through a temperature gradient induced by an infrared laser will be captured and quantified using fluorophores attached to the target protein. When a compound binds to the protein, the degree of changes in thermophoresis reflects the ligand-protein interaction. We found that incubation of NT647-labelled TOP2A(431-1193) with T638 resulted in dose-dependent shifts of thermophoresis. The estimated Kd from this direct TOP2–T638 binding is about 87 ± 40 µM. In contrast, when enzalutamide [35], a known AR binder and inhibitor, was used as a negative control in this experiment, it showed no alteration of thermophoresis of TOP2A protein. Interestingly, merbarone [36] is also a catalytic TOP2 inhibitor that had been hypothesized to bind the DNA binding domain of TOP2 but had not been demonstrated by a biochemistry assay [37,38]. We observed no interaction between merbarone and TOP2A(431-1193). These results together confirmed that T638 directly interacts with TOP2 protein to exert its inhibitory effects. It also indicated that the MOA of T638 is different from merbarone.

### 3.5. T637 and T638 Block TOP2 Proteins from Interacting with DNA

**Figure 5:**
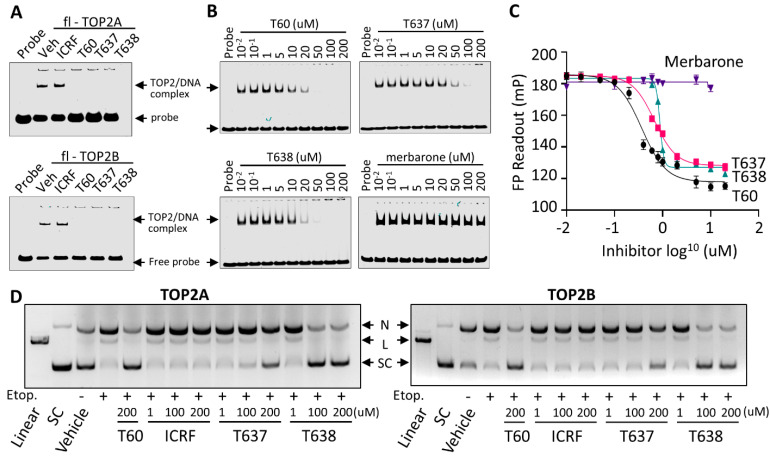
T637 and T638 block TOP2–DNA interaction and prevent TOP2 mediated DNA cleavage. (**A**) EMSA was performed by purified full-length TOP2A or TOP2B proteins at a contraction of 300 nM with IRDay700 labeled 5ZRF DNA oligo at a concentration of 100 nM in the presence of −/+200 µM of ICRF193, T60, T637, and T638. (**B**) EMSA was performed by incubating 2 uM of purified human TOP2A(431-1193) with 300 nM of IRDay700 labeled 5ZRF DNA oligo in the presence of −/+T60, T637, T638, or merbarone. (**C**) FP assay was performed by incubating 500 nM of the purified human TOP2A(431-1193) with 1 nM of 3’ 6-FAM labeled 5ZRF DNA oligo in the presence of 0.01–200 µM of T60, T637, T638, or merbarone. Reactions were subjected to fluorescence emission of 485 nm and excitation of 535 nm, which were detected by using the Multimode Microplate Reader (Infinite F500). (**D**) DNA cleavage assays were performed using full-length human TOP2A and TOP2B incubated with the supercoiled pHOT plasmid in the presence of 200 µM of etoposide plus 1, 100, or 200 µM of ICRF193, T60, T637, and T638. Reactions were separated on a DNA gel with ethidium bromide. All experiments were repeated three times, and one representative image was shown. Note: N, nicked DNA; L, linear DNA; and SC, supercoiled DNA. Full Website Blot Figures are shown in the Supplementary Materials.

To further define the MOA of T638, we have applied three biochemistry techniques. First, we used EMSA to show that T60, T637, and T638, but not ICRF193, block purified full-length TOP2A and TOP2B proteins from forming a complex with DNA oligo (Figure 5A). When purified TOP2A(431-1193) containing the DNA binding domain of TOP2A was used to repeat the EMSA assays, we found that T60, T637, and T638 inhibited TOP2A–DNA interaction dose-dependently, with T638 presenting the strongest effects to block TOP2A–DNA interaction (Figure 5B). In contrast, merbarone did not affect the formation of the TOP2–DNA complex, further confirming that T637 and T638 have a different MOA from merbarone to inhibit TOP2. Second, we applied FP assays in which purified TOP2A(431-1193) was incubated with DNA oligo labeled with NT474 dye. The rate of fluorophore polarization reflects TOP2A–DNA interactions. We found that T60, T637, and T638 reduced the TOP2A–DNA interaction in a dose-dependent manner (Figure 5C), and merbarone did not affect TOP2A–DNA interaction. Lastly, we used DNA cleavage assays to demonstrate that T638 is a catalytic TOP2 inhibitor. When TOP2A or TOP2B enzymes were incubated with supercoiled plasmid DNA, they were shown to bind and then induce DNA cleavage in the presence of etoposide, a TOP2 poison inhibitor (Figure 5D). ICRF193 is a catalytic TOP2 inhibitor that acts to stabilizes the noncovalent TOP2–DNA complex after etoposide induces the TOP2–DNA cleavage complex. It did not prevent etoposide-induced DNA cleavage. In contrast, T637 and T638 did prevent etoposide-induced DNA cleavage, and T638 showed stronger activity than T637. In summary, results from all three assays demonstrated that T638 has a different MOA from merbarone and ICRF193. T638 blocks TOP2 from interacting with DNA and thereby prevents the formation of the TOP2–DNA cleavage complex, and T638 is a catalytic TOP2 inhibitor.

### 3.6. T637 and T638 Inhibit Cancer Cell Growth and Have Limited Genotoxicity

**Figure 6:**
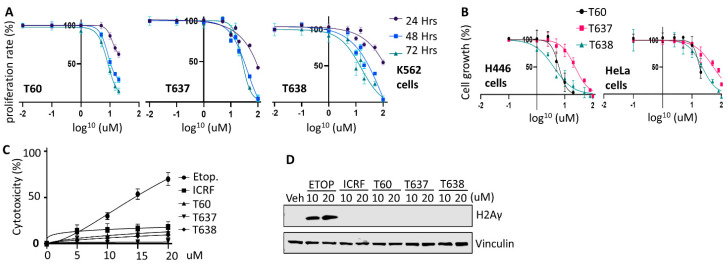
T638 inhibits cancer cell proliferation with low cytotoxicity. (**A**) K562 cells were treated with 0, 0.5, 1, 5, 10, 15, and 20 uM of T60 or 0, 0.5, 1, 5, 10, 15, 20, 50, and 100 uM of T637 and T638 for 24, 48, or 72 h. Cell proliferation rates were measured by MTS assays, and results were calibrated to the vehicle treatment as 100%. (**B**) NCI-H446 small cell lung cancer line and Hela cervical cancer line were treated with 0, 0.5, 1, 5, 10, 15, and 20 uM of T60 or 0, 0.5, 1, 5, 10, 15, 20, 50, and 100 uM of T637 and T638 for five days. Cell growth rates were measured by using Incucyte^TM^. (**C**) K562 cells were treated with 0, 5, 10, 15, and 20 uM of T60, T637, or T638 for 48 h. Cytotoxicity was evaluated by measuring the LDH levels from the culture media. (**D**) K562 cells were treated with the vehicle or 10–20 µM of etoposide, T60, T637, or T638 for 4 h. Immunoblotting measured p-H2AXγ levels in cells with vinculin as a loading control. Full Website Blot Figures are shown in the Supplementary Materials.

Next, we tested the inhibitory effects of T637 and T638 on cell lines derived from different cancer types. In the K562 leukemia cells, T60, T637, and T638 treatments resulted in a dose-dependent inhibition of cell growth. Because T60 started to precipitate when its concentration reached 50 uM in culture media with growing cells inside, the maximum dose we safely applied to cells was 20 uM, and the maximum cell proliferation inhibition that can be achieved is ~38% after 24 h, and 71 and 86% after 48 and 72 h, respectively (Figure 6A). However, improved solubility of T637 and T683 allowed us to be able to treat cells up to 100 uM. The maximum cell proliferation inhibition can be 100% after 48 and 72 h. Consistent results were also obtained from experiments using the small cell lung cancer line NCI-H446 and cervical cancer line Hela (Figure 6B). T638 can achieve 100% cell growth inhibition at the concentration of 100 uM.

To evaluate whether T637 and T638 cause any genotoxic effects similar to etoposide, we have treated K562 cells with increasing doses of etoposide, T60, T637, and T638. Etoposide causes DNA damage inside the cells, and thereby is toxic to cells and causes cell death. During this procedure, a cytoplasmic protein, lactate dehydrogenase (LDH), will be released into cell culture media when the cell membrane is broken. This etoposide-mediated cytotoxicity is in a dose-dependent manner (Figure 6C). It can reach up to 70% cytotoxicity when K562 cells were treated with 20 uM etoposide for two days. However, these effects were not observed when cells were treated with T60, T637, or T638. Furthermore, T637 and T638 do not cause intracellular DNA damage as etoposide does, which was reflected by the phospho-H2AXγ protein levels (Figure 6D). These results were consistent with the MOA of T637 and T638 in that they interrupt TOP2A and TOP2B proteins from recognizing their DNA substrates and forming the TOP2–DNA cleavage complex. Together, these results showed that both T637 and T638 can inhibit cancer cell growth but do not induce DNA damage and cytotoxicity.

## 4. Discussion

This study reports the design of new derivatives based on the lead compound T60 to improve its solubility and preserve its metabolic property and inhibition efficacy to TOP2 activity and cancer cell proliferation. We report here that one successful derivative compound, T638, has improved pharmacokinetic profile and sustained target potency to allow maximal tumor suppression effects.

It has been estimated that about 75% of the drug candidates from the pharmaceutical industry have poor solubility and are classified to Biopharmaceutical Classification System (BCS) classes II and IV [39]. Poor solubility creates challenges for not only in vitro and in vivo drug discoveries, but also drug formulation and delivery to reach required concentrations when testing tumor suppression effects. Our previous studies had shown that the lead compound T60 had reasonable metabolic stability and strong potency, but poor solubility, which prevents us from detecting its maximum inhibition to cancer cell growth. To solve this problem, we have modified the parent scaffold of T60 by adding various moieties on ring 2. Two compounds have shown significantly increased solubility in both PBS and cell culture media (Figure 1). When treating cells with up to 100 uM of T638, we can reach 100% tumor cell suppression.

It has always been challenging to perform medicinal chemistry to balance drug target potency with optimal ADMET and physiochemical properties so that adequate drug exposure can be achieved. This is the case of T637, which has improved solubility but reduced microsomal stability and the potency to block TOP2. In contrast, T638 has enhanced solubility which is accompanied by a significant improvement of microsomal stability (Figure 1) and potency to block TOP2 activity shown in DNA relaxation assays (Figure 2). The increased activity of T638 could be attributed to the formation of an additional hydrogen bond between the amine functional group of ring 2 and Asn851 of TOP2. This is in agreement with our previous findings that derivatives of T60 with modifications on ring 2 exhibited an increased activity [24]. According to our structural modeling, these functional groups can be accommodated in the adjacent cavity within the docking pocket. Thus, this study represents a successful application of an SAR-guided drug optimization approach to enable the lead compound to have increased solubility and enhanced affinity to its protein target.

We report that T638 has a different MOA from other catalytic TOP2 inhibitors, including merbarone and ICRF193. Merbarone is deemed as a catalytic TOP2 inhibitor that blocks TOP2 from inducing DNA cleavage [37]. However, it profoundly causes DNA damage and, thereby, genotoxic effects to cells, arguing whether it is an authentic catalytic TOP2 inhibitor [40,41]. Although some in silico studies had predicted that merbarone binds TOP2 at the region next to the docking pocket of T60 [38], our MST assays do not support that merbarone directly interacts with the DNA binding domain of TOP2 proteins, and our EMSA and FP assays also indicated that merbarone does not interfere with the TOP2–DNA complex (Figure 5). ICRF193 is another representative catalytic TOP2 inhibitor. It traps the TOP2–DNA non-covalent complex, which is formed after TOP2-mediated DNA cleavage (Figure 5). Our studies showed that T638 has an MOA different from both merbarone and ICRF193. T638 is a TOP2 binder but does not intercalate with DNA. Instead, it prevents TOP2 from interacting with DNA and, thereby, does not induce DNA damage (Figure 5D); as such, it has limited genotoxicity (Figure 6).

## 5. Conclusions

In conclusion, we have developed a new T60 derivative, T638, that has improved pharmacokinetic properties and enhancement of inhibitory efficacy to TOP2 and cancer cell growth. T638 has the potential to be further developed into anti-cancer therapy for multiple types of cancers, including, for example, prostate cancers.

## 6. Patents

Patents on T60 and its derivatives have been filed (PCT/62/938,902) or are under application. They can be made available to researchers after standard Material Transfer Agreement (MTA) implementation with the University of British Columbia.

## Data Availability

The data presented in this study are available in this article and Supplementary Materials.

## Supplementary Materials

The following are available online at https://www.mdpi.com/article/10.3390/cancers13153675/s1, Supplementary Materials and Methods and the full Website Blot Figures.
