# Supplementary figures and images for: Optimization of New Catalytic Topoisomerase II Inhibitors as an Anti-Cancer Therapy
Source: Cancers (Basel). 2021 Jul 22;13(15):3675. doi: 10.3390/cancers13153675 (PMC8345109; doi:10.3390/cancers13153675)

Figure 2

A

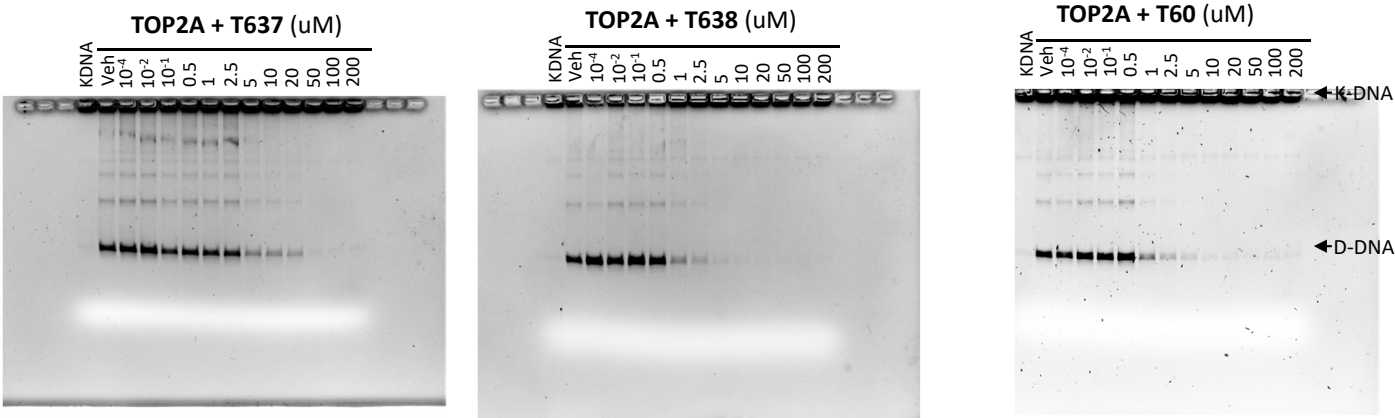

B

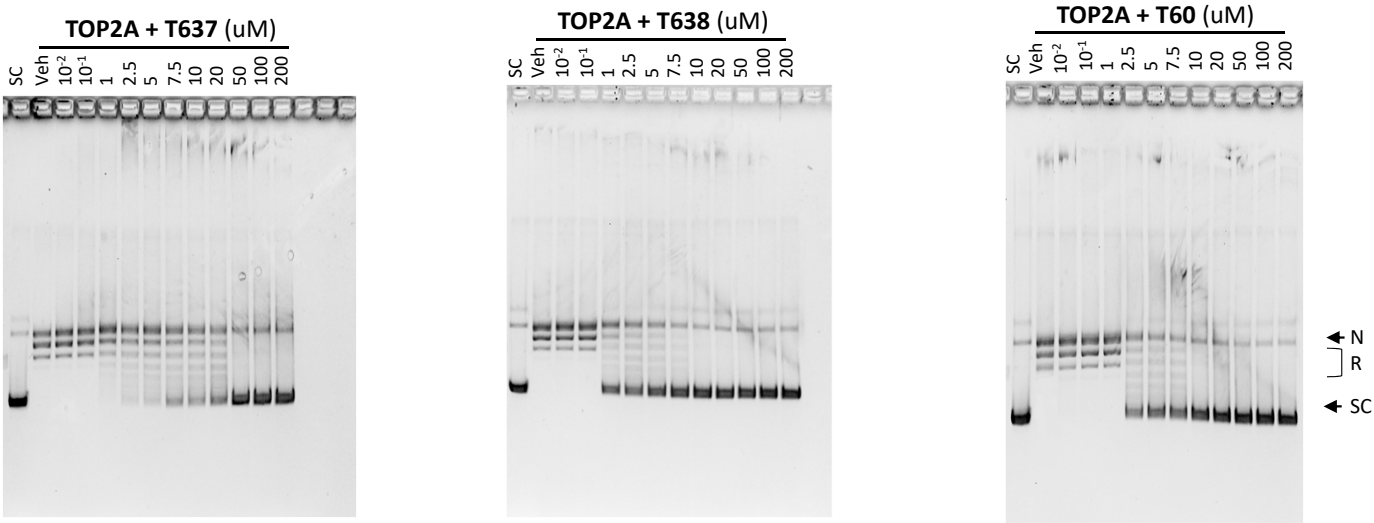

Figure 3

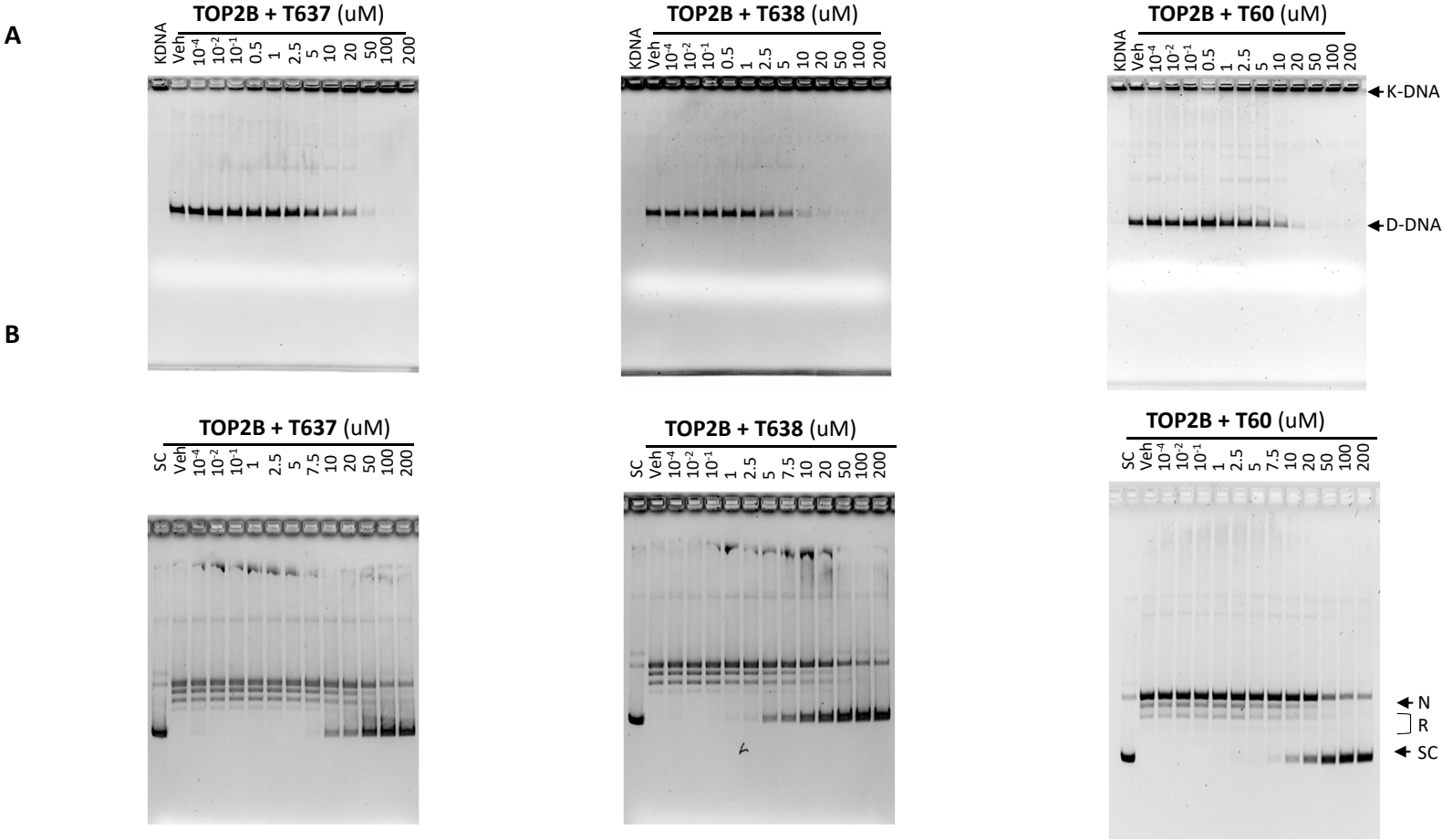

Figure 5

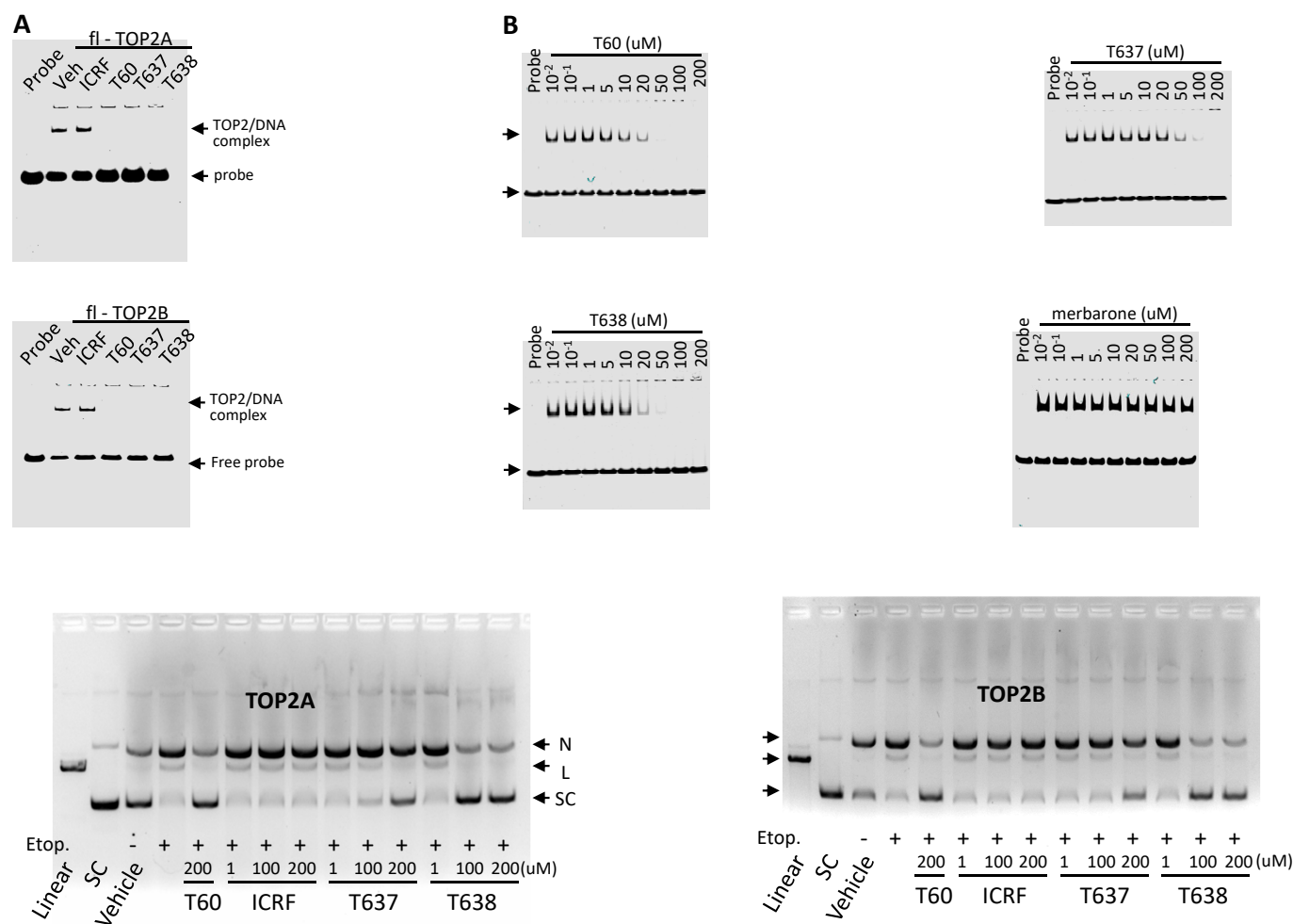

Figure 6

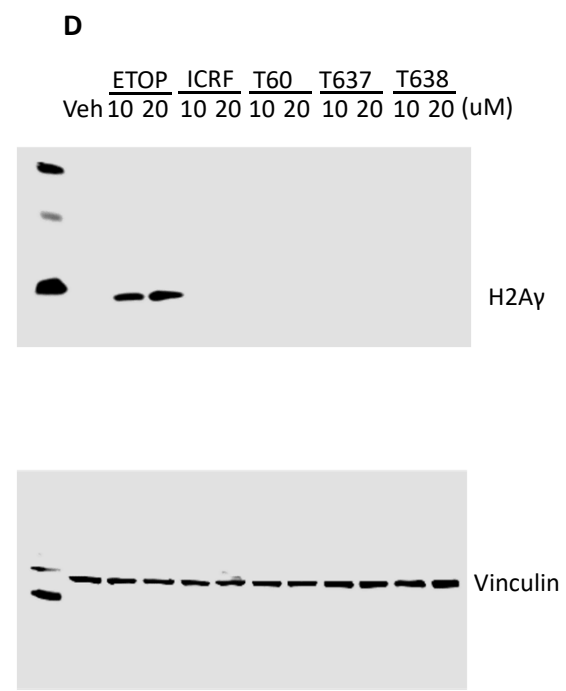

Supplement: Supplementary file 1 [file cancers-13-03675-s001.zip › cancers-1267856-original-images.pdf]
